# Clinical Outcomes of XEN45^®^-Stent Implantation after Failed Trabeculectomy: A Retrospective Single-Center Study

**DOI:** 10.3390/jcm12041296

**Published:** 2023-02-06

**Authors:** Constance Weber, Sarah Hundertmark, Michael Petrak, Elisabeth Ludwig, Christian Karl Brinkmann, Frank G. Holz, Karl Mercieca

**Affiliations:** 1 Department of Ophthalmology, University of Bonn, 53127 Bonn, Germany; 2 Department of Ophthalmology, Dietrich-Bonhoeffer Hospital, 17022 Neubrandenburg, Germany

**Keywords:** glaucoma surgery, MIGS, trabeculectomy, XEN, fibrosis

## Abstract

Background: The implantation of a collagen gel micro-stent (XEN45^®^) as a minimally invasive form of glaucoma surgery (MIGS) after a failed trabeculectomy (TE) may be an effective option with few risks. This study investigated the clinical outcome of XEN45^®^ implantation after a failed TE, with follow-up data of up to 30 months. Materials and Methods: In this paper, we present a retrospective review of patients undergoing XEN45^®^ implantation after a failed TE at the University Eye Hospital Bonn, Germany, from 2012 to 2020. Results: In total, 14 eyes from 14 patients were included. The mean follow-up time was 20.4 months. The mean time duration between the failed TE and XEN45^®^ implantation was 110 months. The mean intraocular pressure (IOP) decreased from 17.93 mmHg to 12.08 mmHg after one year. This value increased again to 17.63 mmHg at 24 months and 16.00 mmHg at 30 months. The number of glaucoma medications decreased from 3.2 to 0.71, 2.0, and 2.71 at 12, 24, and 30 months, respectively. Conclusions: XEN45^®^ stent implantation after a failed TE did not lead to an effective long-term decrease in IOP and glaucoma medications in many patients in our cohort. Nevertheless, there were cases without the development of a failure event and complications, and others in whom further, more invasive surgery was delayed. XEN45^®^ implantation in some failed trabeculectomy cases may, therefore, be a good option, especially in older patients with multiple comorbidities.

## 1. Introduction

Trabeculectomy (TE), a technique that was first developed in 1968, is still considered by many as the gold standard filtration surgery for glaucoma [1]. Although very effective, it may become necessary to perform a needling procedure or a formal revision when bleb filtration fails over the course of time [2,3,4,5,6,7]. Minimally invasive glaucoma surgeries (MIGS) were developed as an alternative to more extensive procedures, such as trabeculectomy. The term encompasses a wide group of less invasive MIGS surgeries, which are more particularly defined by an ab interno intracameral micro-incisional approach [8,9,10]. The XEN45^®^ Gel Stent (Allergan Inc., Irvine, CA, USA) can be considered a type of MIGS device, albeit with the added invasiveness of sub-conjunctival filtration and the need for antimetabolite administration [11,12]. This hydrophilic stent has been shown to be quite effective in IOP reduction while having a lower complication rate compared with more aggressive surgeries, such as trabeculectomy [13,14].

In cases with failed trabeculectomy, glaucoma drainage devices or revision surgery of the failed trabeculectomy site can lead to the further lowering of IOP [15,16,17]. However, these interventions are associated with risks such as postoperative hypotony, postoperative wound leakage, and corneal decompensation [18]. A few studies have described the outcomes of XEN45^®^ gel stent implantation as a therapy modality after a failed trabeculectomy, showing that it can be an effective option for certain patients [19,20,21]. However, there are no current studies showing long-term follow-up data beyond 12 months.

## 2. Materials and Methods

### 2.1. Patients

All the medical records of patients who underwent XEN45® implantation for chronic open-angle glaucoma, pseudo-exfoliation glaucoma, and neovascular glaucoma at the Department of Ophthalmology at the University Hospital of Bonn, Germany, from 2012 until 2020 were reviewed retrospectively. All patients received standalone XEN45^®^ implantation without combined cataract surgery. Only those patients who had received a XEN45^®^ implantation after a failed trabeculectomy and with complete follow-up data over 12 months were included for further analysis. Patients who had undergone surgery without an earlier trabeculectomy and those with incomplete follow-up data were subsequently excluded. 

All patients underwent a full ophthalmic examination upon presentation, including an assessment of best-corrected visual acuity (BCVA) using Snellen charts (converted to logMAR for statistical evaluation), IOP measurement via Goldmann applanation tonometry, slit-lamp biomicroscopy, fundus biomicroscopy, and a visual field test, using the Humphrey 24-2 (Carl Zeiss Meditec, Inc., Dublin, CA, USA) for a visual field test. The documented data included gender, age, glaucoma type, BCVA, preoperative clinical features (such as IOP and visual field test results), and detailed follow-up information regarding BCVA, IOP, visual fields, complications, and postoperative glaucoma medication. Patients were censored for further analyses from the point of any additional glaucoma surgery. Patients received an additional glaucoma operation if the IOP was insufficiently controlled after XEN45® implantation, despite maximal therapy and visual field progression.

The failure of XEN45^®^ implantation surgery was defined as an IOP either below 6 mmHg or over 21 mmHg, measured over two visits paid at least 3 months after surgery; the need for additional glaucoma surgery was established for persistently raised IOP and/or in cases resulting in the loss of light perception. All analyses were conducted on a de-identified data set. A waiver from the local Ethics Committee was granted, due to the retrospective nature of the study. The study protocol conformed to the ethical guidelines of the 2000 Declaration of Helsinki, as reflected in the a priori approval granted by the institution’s Human Research Committee. 

### 2.2. Statistical Analysis

Statistical analysis was performed with SPSS Statistics version 27.0.0 (IBM Corporation, New York, NY, USA). Time-dependent survival probabilities were estimated with the Kaplan–Meier method.

## 3. Results

In total, 14 patients were included in this study. The mean age was 71 years. After exclusions, 5 women (35.7%) and 9 men (64.3%) were included. The mean BCVA, measured preoperatively, was 0.47 logMAR. Thirteen patients (92.9%) were pseudophakic. Further characteristics of our patient cohort are described in Table 1. 

The mean follow-up time was 20.4 months, with a maximum follow-up time of 34 months (12–34 months). Eight patients (57.1%) had a diagnosis of primary open-angle glaucoma (POAG), while 5 patients (35.7%) had pseudo-exfoliative (PEX) glaucoma. The preoperative mean IOP was 17.93 mmHg, with a maximum of 25 mmHg and a minimum of 11 mmHg. The mean maximum IOP obtained from each patient’s medical history was 22.27 mmHg (16–35). Preoperatively, patients were on a mean number of 3.2 topical glaucoma medications. The majority of patients (7; 50%) were taking 4 pressure-lowering agents. Almost all patients (13; 92.9%) were not on additional systemic acetazolamide therapy. The mean vertical cup-to-disc ratio was 0.78. The mean deviation (MD), measured preoperatively with a Humphrey visual field analysis (24-2), was −13.75 dB and the pattern standard deviation (PSD) was 8.6 dB.

The mean duration between the operative date of the failed trabeculectomy and the XEN45^®^ implantation surgery was 110 months, with a maximum of 270 months and a minimum of 36 months. During the postoperative course, 3 patients (21.4%) underwent an additional glaucoma procedure. Of these 3 patients, one patient underwent trans-scleral cyclo-diode laser treatment, one patient underwent trabectome surgery, and one patient underwent *PreserFlo*^®^ Microshunt implantation. The mean time period between XEN45^®^ gel implantation and additional glaucoma surgery was 10.33 months (range: 1–22 months; SD: 10.69).

**Figure 1:**
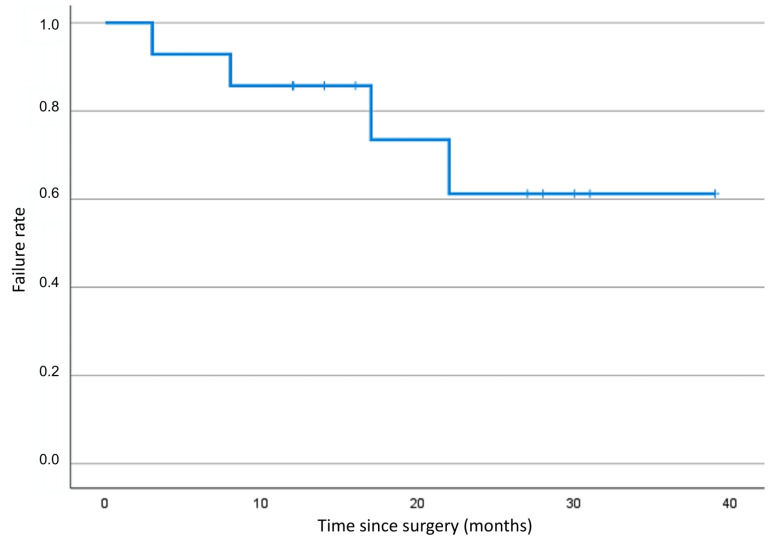
Kaplan–Meier curve depicting the failure rate after XEN45^®^ implantation surgery.

Of all 14 patients, 4 patients (28.6%) developed a failure event (Figure 1).

Failure criteria were applied, as explained above. As aforementioned, 3 patients of the 4 (75.0%) failed due to additional glaucoma surgery, and 1 patient (25%) failed due to persistent high IOP levels over 21 mmHg, recorded over two visits (Table 2).

Five patients (35.7%) developed complications during the postoperative course; two of these had more than one complication. One patient experienced a hyphema immediately after surgery and one patient exhibited corneal oedema. These complications eventually resolved without the need for additional interventions. 

One patient showed a numerical hypotony during the first days after surgery (IOP < 6 mmHg, with no sign of choroidal detachment).

Two patients underwent revision surgery of the XEN45^®^ implant, with the repeated application of Mitomycin C; one patient underwent partial removal of the XEN45^®^ implant (Table 2). Both patients were revised two weeks after surgery. These patients had an IOP of 5 mmHg and 10 mmHg, respectively, during the week after the revision. These IOP values went up to 12 and 14 mmHg, respectively, 10 months post revision, and 16 and 17 mmHg, respectively, 22 months post revision. Both patients were started off with pressure-lowering drops at 3 months after revision with one and three glaucoma agents, respectively. 

On the first day after surgery (*n* = 14), the IOP decreased to a mean IOP of 9.86 mmHg, with an IOP drop of 40% in comparison to the preoperative IOP. The number of IOP-lowering drops was considerably lower than before surgery (0.29). One week after surgery, the results were comparable to the first day after surgery (*n* = 14). The IOP was 10.63 mmHg and the mean number of pressure-lowering eye drops was 0.5. During both time periods, the BCVA had decreased.

**Figure 2:**
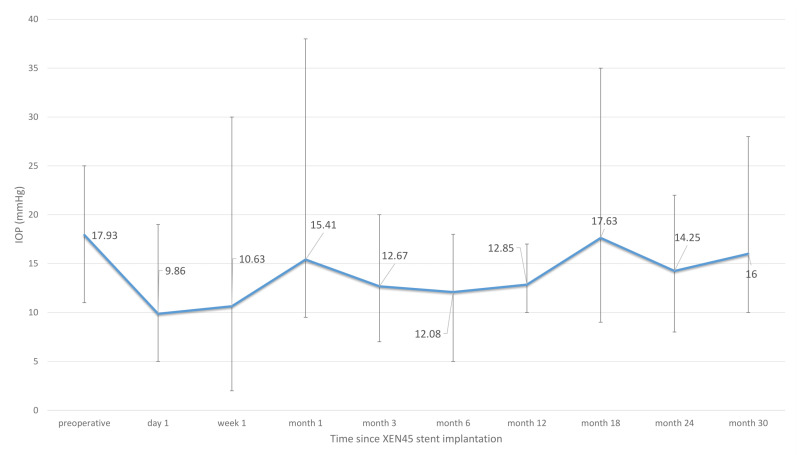
The IOP values (bars represent range with minimum and maximum) over time for all patients with XEN45^®^ stent implantation after a failed trabeculectomy.

During the postoperative course, the IOP showed an increase in IOP values between 12 and 17 mmHg. One month after surgery (*n* = 14), the IOP was 15.41 mmHg; 3 months after surgery, it was 12.67 mmHg (*n* = 14), and six months after surgery, it was 12.08 mmHg (*n* = 14). One year after surgery, the follow-up data were complete for all 14 patients and showed a mean IOP of 12.08 mmHg. In comparison to the preoperative value, the mean IOP had decreased by 4 mmHg. However, the mean IOP had further increased to 17.63 mmHg at 18 months (*n* = 9), then changed to 14.25 mmHg at 24 months (*n* = 7), and to 16.00 mmHg at 30 months after surgery (*n* = 7) (Figure 2). 

When only the 7 patients with a follow-up time of 24 months were included in the analysis, the mean preoperative IOP was 19.25 mmHg at month 6, 13.63 mmHg at month 12, 17.63 mmHg at month 24, and 16 mmHg at month 30. 

**Figure 3:**
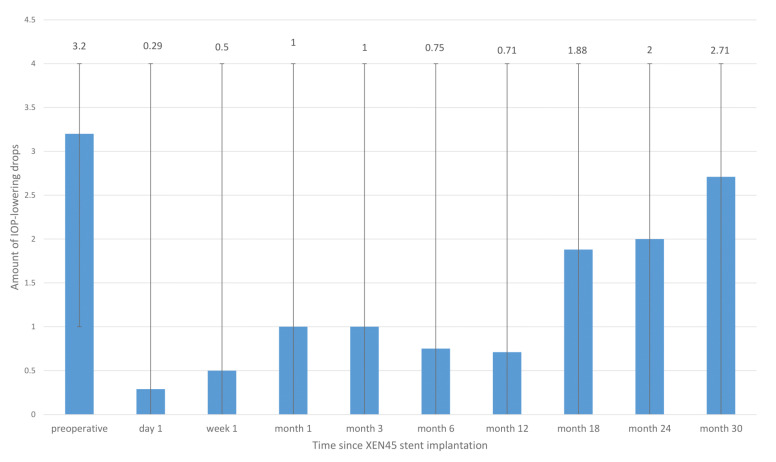
Number of IOP-lowering agents (bars represent a range with the minimum and maximum) over time for all patients undergoing XEN45^®^ stent implantation after a failed trabeculectomy.

The amount of pressure-lowering eye drops was 1.0 at one month (*n* = 14), 1.0 at 3 months (*n* = 14), 0.75 at 6 months (*n* = 14), and 0.71 at one year after surgery (*n* = 14). Compared with the preoperative amount of 3.2 drops, the number was considerably lower at one year after surgery (Figure 3).

Nevertheless, the amount increased again, with a mean of 2.0 drops at two years (*n* = 7) and 2.71 drops at 30 months after surgery (*n* = 7). 

Overall, the BCVA showed some deterioration during the time period after surgery, from 0.52 logMAR, preoperatively, to 0.84 logMAR one week after surgery (*n* = 14), 0.69 logMAR after 6 months (*n* = 14), 0.72 logMAR after 12 months (*n* = 14), 0.82 logMAR after 24 months (*n* = 7), and 0.84 logMAR 30 months (*n* = 7) after surgery.

The mean preoperative cup-to-disc-ratio (CDR) was 0.78 (0.5–0.99) and showed a slight deterioration during the postoperative course, with 0.81 (0.5–0.99) after 24 months (*n* = 7) and 0.81 (0.5–0.99) after 30 months (*n* = 7). 

The visual field test results showed a mean deviation of −15.75 dB at six months (*n* = 14), −16.92 dB at one year (*n* = 13), −15.46 dB at two years after surgery (*n* = 6) and −16.43 dB at 30 months (*n* = 6). In comparison to a preoperative value of −13.75 dB, these results showed a slight progression in the visual field tests. However, when comparing the pattern standard deviation with a preoperative value of 8.6 dB to 9.5 dB at 6 months (*n* = 14), 7.2 dB at 1 year (*n* = 13), 9.9 dB at 2 years (*n* = 6) and 10.1 dB at 30 months (*n* = 6), the visual field defects showed only a discrete progression. 

## 4. Discussion

To date, trabeculectomy remains the mainstay of surgical treatment for primary open-angle glaucoma for many glaucoma surgeons worldwide. Although the techniques have been refined over time, and scarring can be managed more effectively by the use of mitomycin C and 5-fluorouracil, failure is still frequent. Gedde and colleagues described a probability of failure for trabeculectomy of 28% during the first three years after surgery. Reasons for failure were inadequate IOP reduction, reoperation for glaucoma, persistent hypotony, or the loss of light perception [22]. Fontana and colleagues even state that only half of the cases achieved long-term low IOP after trabeculectomy [23].

The feasibility of using a less invasive glaucoma procedure after a failed trabeculectomy, such as a XEN45^®^ implant, has only been described in a few studies, but, to our knowledge, these cases have only a limited follow-up. 

To the best of our knowledge, our study shows the longest periods of follow-up results available until now for XEN45^®^ stent implantation after a failed trabeculectomy. This aspect is especially important because the efficacy of a glaucoma procedure can only be fully evaluated after a long follow-up period. Our study contained a follow-up time of 20.4 months (12–34 months). Data were complete for every patient of our cohort after one year, were still complete for half of the patients after 24 months, and complete for 7 patients after 30 months. The other half were lost to follow-up or were censored due to failure events. Karimi and colleagues performed a retrospective study with 17 patients in 2018, but they only included follow-up data for 12 months [19]. Bormann and colleagues performed examinations of 31 eyes of 28 patients who received a XEN45® after an insufficient trabeculectomy and included follow-up data for up to twelve months [20]. Düzgün and colleagues demonstrated a mean follow-up period of 14.2 months [21]. 

We included 14 patients with a preoperative IOP of 17.93 mmHg, with a range from 11 mmHg to 25 mmHg. One patient had a preoperative IOP of 11 mmHg, which is considered quite low for listing this patient for surgery. However, this patient had been prescribed an intake of acetazolamide and the maximum amount of pressure-lowering medication, with an even lower target IOP.

In our cohort, the IOP was lowered to an IOP of 12.08 mmHg on average (preoperative measurement: 17.93 mmHg) and the usage of pressure-lowering agents decreased to a mean of 0.71 (preoperative measurement: 3.2 drops). After a time interval of one year, in comparison with our study, the aforementioned studies reported effective IOP-lowering after XEN45^®^ stent implantation. Düzgun et al. described 14 patients who received XEN45^®^ in the inferonasal quadrant, who showed an IOP-reduction of 49.3% (from 24.14 mmHg to 12.23 mmHg) and a reduction in eyedrops from 3.71 to 1.31, on average [21]. Karimi and colleagues performed a retrospective study with 17 patients in 2018, showing that XEN45^®^ reduced the IOP from 21.5 to 13.6 mmHg and the number of pressure-lowering medications from 2.8 to 1.0 after 12 months [19]. Bormann and colleagues stated that XEN45^®^ reduced the IOP from 23.5 to 18.0 at 12 months, postoperatively [20]. All these studies, including our own, demonstrate an efficient reduction in IOP and in the number of pressure-lowering eyedrops at one year post-XEN45^®^ stent implantation after a failed trabeculectomy, and concluded that XEN45^®^ could be a good method to overcome failed trabeculectomies with minimally invasive surgery. 

The above-referenced studies did not report further outcomes from other time points. To the best of our knowledge, there are no other studies presenting data on 2-year results for XEN45^®^ implantation after a failed trabeculectomy. Karimi and colleagues even conclude in their list of study limitations that a follow-up beyond 12 months would have been better to evaluate the procedure’s true efficacy after a failed TE. 

In our study, we were able to maintain a connection with half of our patients for follow-up data after 30 months. The outcome after two years and 30 months showed that, unfortunately, the IOP-lowering and drug-reducing effect decreased, and, in some patients, had even reached a similar level as preoperatively. The mean IOP increased from 12.08 mmHg at month 12 to 17.63 mmHg at month 18, 14.25 mmHg at month 24, and 16.00 mmHg at month 30 after surgery. The IOP drop between month 18 (*n* = 9) and month 24 (*n* = 7) might be due to the exclusion of one patient who had higher IOP values and was subsequently excluded from analysis because of a failure event, while the other patient with rather high IOP values did not have further follow-up appointments. However, it must be taken into account that follow-up data at 24 and 30 months after surgery were not available for every patient and that the results can be affected by selection bias. To rule this out, we compared the IOP when only including those seven patients with an available follow-up time of 24 months (preop: 19.25, month 12: 13.63 mmHg, month 24: 17.63 mmHg). This subgroup analysis showed that these patients had a higher preoperative IOP than the average group and, thus, the IOP remained lower at month 24 than preoperatively, but was still considerably higher than after a year. The long-term follow-up data included in our study shows that XEN45^®^ stent implantation first leads to an IOP reduction during the first year, with an increasing IOP during the second year. This is most probably due to fibrosis formation around the subconjunctival portion of the stent. 

Since one of the main reasons for failure in TE is bleb fibrosis, and since the XEN45^®^ follows the same principle of subconjunctival filtration, it seems logical that a XEN45^®^ stent would suffer from the same problem and, thus, would not be effective after a longer time period. Furthermore, one assumes that a failed trabeculectomy would mean that that particular patient would also be at higher risk of subconjunctival scarring and bleb failure, both pre-trabeculectomy and also even more so after having already undergone a bleb-modifying operation. In these cases, a glaucoma drainage device (GDD), where a tube drains into the untouched post-equatorial subconjunctival space, is considered by many to be the preferred therapy option after a failed TE, especially if post-TE interventions, such as bleb needling and/or repeated antimetabolite application, have been unsuccessful in restoring bleb function. However, tube surgeries can also pose certain risks, which a XEN45^®^ stent avoids to a significant degree. A GDD procedure is usually more complex and time-consuming and will more often result in major complications, especially with regard to persistent postoperative hypotony, corneal endothelial cell loss, and transconjunctival erosions. In addition, general anesthesia is more often necessary for GDD than it is for XEN45^®^. 

In concordance with the other studies described earlier, XEN45^®^ stent implantation is a safe procedure with mostly minor complications. In our study, 5 patients (35.7%) developed complications; however, most of these were resolved without the need for additional interventions. One patient (7.1%) had a hyphema immediately after surgery, one patient (7.1%) developed numerical hypotony, and one patient (7.1%) exhibited corneal oedema, all of these complications resolving themselves without further interventions. Bormann and colleagues reported that 4 eyes (13%) developed choroidal detachment, due to postoperative hypotony, and 4 eyes (13%) developed a subconjunctival hemorrhage. Düzgün and colleagues reported that 5 eyes (35.7%) developed intracameral hemorrhage and 3 eyes (21.4%) developed postoperative numerical hypotony [21]. Both complications were resolved without additional procedures. The authors did not report any severe complications and there were no cases of vision loss, bleb infection, or stent exposure in their study. The most frequent complication described in the study published by Karimi et al. [19] was the occurrence of numerical hypotony (IOP < 6 mmHg), which resolved itself, as in our patients, without intervention within a week. All the above-mentioned studies, including ours, demonstrate that XEN45^®^ stent implantation is a safe procedure with a small risk of major complications.

However, XEN45^®^ stent implantation may quite often be followed by small postoperative interventions and revision procedures to maintain its IOP-lowering effect. In our study, two patients (14.2%) had to undergo revision surgery of the implant with the repeated administration of Mitomycin C, one patient (7.1%) underwent needling, and one patient (7.1%) underwent a partial removal of the XEN45^®^ implant. Bormann and colleagues reported similar results, with 10% needing an open revision of the conjunctiva, while 9 eyes (29%) received postoperative needling. Düzgün and colleagues reported that six eyes (42.8%) required postoperative bleb needling during the postoperative course. Karimi et al. reported that postoperative bleb intervention was performed in 9 eyes (52.9%), with a mean of 2.4 postoperative bleb needling/injections. Most patients required one of these interventions after one month. 

This relative lack of risk is why XEN45^®^ stent implantation should still be considered in selected cases after a failed trabeculectomy, especially in older people with multiple comorbidities, as it offers a less invasive and quicker approach to lowering IOP for a period of time, albeit with the risk of an increase during the postoperative course. Additionally, failed XEN45^®^ rescue surgery does not preclude GDD surgery in the future, which continues to be a treatment option later in due course. 

To the best of our knowledge, our study is the only one that includes data on visual field progression. Our 2-year results showed a slight progression of the mean deviation. The visual field test results showed a mean deviation of −13.75 dB, preoperatively, and −15.46 dB at two years after surgery. The pattern standard deviation showed a similar development from 8.6 dB, preoperatively, to 9.9 dB at 2 years. One must consider that these results are partially biased due to the selection bias inherent in the retrospective nature of this study. On top of that, in the German healthcare system, patients are usually referred to tertiary care centers in the case of insufficient pressure control or the progression of their visual fields, whereas adequately controlled patients are followed up by their respective local ophthalmologists. 

The slight progression in visual field results shows that there might be another downside to performing a XEN45^®^ implantation after a failed TE. In these patients, after another rescue operation is performed, an ophthalmologist might feel safer about visual fields and IOP control and may be rather conservative toward more extensive surgeries in cases where the XEN45^®^ fails as well. Eventually, ineffective treatment with XEN45^®^ might impede or delay other, more appropriate, treatment options in the longer term. In addition to that, patients might be hesitant to have another operation since they have only just been operated upon and would not be able to comprehend the relative implications of less and more invasive surgery. It is therefore imperative that after a failed trabeculectomy, one must consider which patients might truly benefit from XEN45^®^ stent implantation. It seems that younger patients with a high risk of scarring might benefit more from alternative procedures, such as GDD surgery.

## Figures and Tables

**Table 1:** Demographics and clinical characteristics of patients undergoing XEN stent implantation after a failed trabeculectomy.

Patient Characteristics	*n* = 14 (%)
**Gender**	
Male/Female	9 (64.3)/5 (35.7)
**Age**	
Mean, median (range, SD)	71.14, 74.50 (51–84; 9.51)
**Glaucoma type**	
Primary open-angle glaucoma	8 (57.1)
Pseudoexfoliation glaucoma	5 (35.7)
Secondary neovascular glaucoma	1 (7.1)
**Follow-up time (months)**	
Mean, median (range, SD)	20.36, 20.50 (12–34; 8.43)
**Time between TE and XEN (months)**	
Mean, median (range, SD)	110.54, 100.00 (36–270; 10.33)
**Acetazolamide**	
Yes/no	1 (7.1)/13 (92.9)
**Number of glaucoma medications**	
0	0
1	2 (14.3)
2	3 (21.4)
3	2 (14.3)
4	7 (50.0)
5	0
**BCVA, measured preoperatively (logMAR)**	
Mean, median (range, SD)	0.52, 0.35 (0–2.3; 0.62)
**IOP preoperatively**	
Mean, median (range, SD)	17.93, 18.00 (11–25; 4.45)
**Maximum IOP without drops in the past**	
Mean, median (range, SD)	22.27, 21.00 (16–35; 4.40)
**Mean deviation (MD) (Humphrey 24-2)**	
Mean, median (range, SD)	−13.75, −15,50 (−1.96–−25.25; 8.13)
**Pattern standard deviation (PSD)**	
Mean, median (range, SD)	8.6, 9.53 (3.1–14.54; 3.79)
**Cup-to-disc ratio (CDR)**	
Mean, median (range, SD)	0.78, 0.80 (0.50–0.99; 0.17)

**Table 2:** Complications and failure after XEN45 stent implantation.

Complications	
Yes/no	5 (35.7)/9 (64.3)
**Complications (3 patients with more than 1 complication)**	
Hyphema	1 (7.1)
XEN-Revision + Mitomycin C	2 (14.2)
Needling	1 (7.1)
Partial XEN removal	1 (7.1)
Corneal oedema	1 (7.1)
Numerical hypotony	1 (7.1)
**Failure yes/no**	
Yes/no	4 (28.6)/10 (71.4)
**Failure reason**	
Loss of visual acuity to levels below light perception	0
Hypotony < 6 mmHg over two consecutive visits	0
Persistent high pressure > 21 mmHg over two consecutive visits	1 (25.0)
Additional glaucoma surgery needed	3 (75.0)
**Time interval since XEN operation (months)**	
Mean (range)	10.33 (1–22)

## Data Availability

All datasets generated during and/or analyzed during the current study are available from the corresponding author on reasonable request.
